# Disordered eating in adolescence: the roles of sensation seeking, weight status, and behavioral difficulties

**DOI:** 10.1186/s40337-026-01620-y

**Published:** 2026-04-26

**Authors:** Marianna Bogner, Ricarda Schmidt, Anja Hilbert, Mandy Vogel, Juliane Ludwig, Wieland Kiess, Tanja Poulain

**Affiliations:** 1 LIFE Leipzig Research Center for Civilization Diseases, Leipzig University, Philipp-Rosenthal-Straße 27, 04103 Leipzig, Germany; 2 Department of Child and Adolescent Psychiatry, Psychotherapy and Psychosomatics, University Hospital Leipzig, Leipzig, Germany; 3 German Center for Child and Adolescent Health (DZKJ), partner site Leipzig/Dresden, Leipzig, Germany; 4 Behavioral Medicine Research Unit, Department of Psychosomatic Medicine and Psychotherapy, University of Leipzig Medical Center, Leipzig, Germany; 5 Department of Women and Child Health, Hospital for Children and Adolescents, Center for Pediatric Research (CPL), Leipzig University, Leipzig, Germany

**Keywords:** Sensation seeking, Disordered eating, Behavioral difficulties, Emotional symptoms, BMI, Sex, Socioeconomic status, Adolescence

## Abstract

**Background:**

Previous studies that have focused primarily on binge eating in young adults have suggested an association between the personality trait sensation seeking and disordered eating. Far less is known about this relationship in adolescents, particularly concerning sensation seeking and eating disorder psychopathology. Thus, the present study investigated the association between sensation seeking and disordered eating, including eating disorder psychopathology and relevant diagnostic symptoms of eating disorders (i.e., binge eating) in healthy adolescents, while also considering relevant sociodemographic variables. Additionally, moderating effects of weight status and behavioral difficulties were explored.

**Methods:**

Four hundred 13- to 15-year-old participants from the LIFE Child study (Leipzig, Germany) provided information on sensation seeking (encompassing novelty- and intensity-seeking), disordered eating, behavioral difficulties, and sociodemographic data. Objective anthropometric measurements (body mass index, BMI) were also taken. Multiple linear regression models were applied to assess associations between sensation seeking and sociodemographic variables, as well as between sensation seeking and disordered eating. BMI and behavioral difficulties were included as moderators for analyses on interactions.

**Results:**

Boys displayed higher levels of sensation seeking than girls, and novelty seeking was positively associated with socioeconomic status. Sensation seeking was positively associated with restraint and overeating. Associations with eating concern, loss of control eating, and binge eating did not remain significant after correction for multiple testing. The association between sensation seeking and disordered eating was stronger in adolescents with a higher BMI, more symptoms of hyperactivity/inattention, more emotional symptoms, and more peer problems.

**Conclusion:**

The results indicate that higher sensation seeking is associated with restraint and overeating in adolescence. Hyperactivity/inattention and internalizing symptoms were shown to moderate associations between sensation seeking and disordered eating, suggesting that they may be promising targets for prevention strategies and interventions.

## Abstract (plain language summary)

Unhealthy eating patterns display a worldwide health concern in adolescence. In the present study we aimed to investigate the association between the personality trait sensation seeking (SS) and disordered eating. Information on SS, eating disorder psychopathology, diagnostic symptoms of eating disorders and behavioral difficulties were gathered from 400 13- to 15-year-old adolescents who participated in the LIFE Child study (Leipzig, Germany). Results showed that SS was positively associated with restraint and overeating. Associations with eating concern, loss of control eating, and binge eating did not remain significant after correction for multiple testing. Moderation analyses revealed that the association between SS and disordered eating was stronger in those with a higher BMI, more symptoms of hyperactivity/inattention, more emotional symptoms, and more peer problems. These moderating effects suggest that intervention strategies addressing hyperactivity and enhancing emotion regulation might be especially beneficial for adolescents with higher levels of impulsivity-related traits, such as SS.

## Introduction

Sensation seeking is a personality trait describing an individual´s “seeking of varied, novel, complex, and intense sensations and experiences, and the willingness to take physical, social, legal, and financial risks for the sake of such experience” [1]. Individuals with high sensation seeking levels usually avoid boredom and pursue unpredictable events and ambiguous situations or experiences [2]. The period of childhood and adolescence is an especially vulnerable phase of changes in personality and behavior, including sensation seeking. Sensation seeking follows an inverted U-shaped pattern [3] with increases from early childhood on [4], peaking at around 19 years [3], and declining through the mid-20s [5] until young adulthood (30 years) [6]. In most studies, male adolescents have shown higher levels of sensation seeking than female adolescents [7–9]. Previous studies have yielded mixed findings regarding the relationship between sensation seeking and socioeconomic status (SES). Whereas Russo et al. found a positive relationship between thrill- and adventure-seeking and SES in 9- to 14-year-old male adolescents [8], other studies have reported no significant associations [4, 10, 11].

In recent decades, research on sensation seeking in adolescence primarily has addressed associations with health-related behavior and development, for example, drug and alcohol use or risky sexual behavior [12–16]. Considering that the behaviors of individuals with drug addictions share similar neurobiological correlates with the addictive-like consumption of appetitive foods [17–19], sensation seeking may play a role in eating disorders as well. Eating disorders display a worldwide health concern of increasing incidence [20], with adolescence being the most critical age of onset [21]. Research on associations between sensation seeking and eating disorder psychopathology in adolescents is scarce. Jansen et al. showed higher sensation seeking in female university students with restrained eating behavior compared with nonrestrained eaters [22]. By contrast, Argyriou et al. found no significant association between sensation seeking and cognitive restraint (regarding eating behavior) in adults from a general population [23]. Diagnostically relevant characteristics of eating disorders include behaviors such as loss of control eating, describing episodes of consuming food with the sense of being unable to stop eating, and binge eating, which refers to episodes of consuming objectively (or subjectively) large amounts while experiencing a lack of control over eating. Compared with control groups, increased sensation seeking levels were found in young women who engaged in weekly binge-eating episodes [24] and those with related eating disorders, including bulimia nervosa and binge-eating disorder [25–27]. Furthermore, young women with bulimia nervosa have reported greater sensation seeking behavior compared with individuals with restricting anorexia nervosa [26, 28, 29], characterized, amongst others, by dietary restraint without engagement in binge eating or purging behavior [25]. Only a few studies have examined the relationship between sensation seeking and disordered eating longitudinally, with findings that have been partially contradictory to those mentioned above. According to Hirvelä et al., sensation seeking at the age of 18 years was weakly associated with later bulimic symptoms in young men, whereas no association could be found for young women [30]. Brown et al. found that, in girls from a community-based sample, lower sensation seeking in childhood significantly predicted binge eating and purging at the age of 14 years [31]. Apart from the longitudinal design, Hirvelä et al. [30] Brown et al. [31] also included boys and male adolescents, in contrast to the studies mentioned above.

Individuals with eating disorders characterized by binge-eating episodes have demonstrated a high comorbidity with lifetime obesity [32]. Compared with adolescents with normal weight, Delgado-Rico et al. identified diminished levels of sensation seeking in adolescents with overweight and obesity [33], whereas Nazarbol et al. found elevated levels of sensation seeking in adolescents with obesity relative to adolescents with normal weight [34]. Consistently, both studies suggest that treatment-seeking adolescents with overweight or obesity show higher levels of emotion-driven impulsivity (positive and negative urgency) and are prone to engaging in excessive food intake when they experience intense affective states [33, 34]. Sensation seeking is widely acknowledged as a dimension of impulsivity, but is not the same construct. Therefore, it is also unclear whether there may be a similar connection here in the sense of “emotion-driven sensation seeking”. A community-based study assessing event-related brain potentials revealed more deficits in emotion regulation in adolescents (aged 10–15 years) with high sensation seeking levels compared with those with low levels [35], independently from weight status.

Regarding general psychopathology, sensation seeking was not associated with internalizing behaviors [36–38] but was positively correlated with externalizing behaviors in youth, such as conduct problems and aggression [39–41]. Regarding hyperactivity/inattention, past studies have yielded mixed findings. Marmorstein found no significant associations between sensation seeking and symptoms of hyperactivity/inattention [36], whereas Dekkers et al. suggested a positive correlation between sensation seeking and attention problems in children and adolescents from a general population [37].

Past studies have often focused primarily on the association between sensation seeking and binge eating, while not taking into account important cognitive aspects of disordered eating behavior, such as restraint and body dissatisfaction. Furthermore, to our knowledge, externalizing and internalizing behaviors have not yet been investigated as moderating variables of associations between sensation seeking and disordered eating in adolescence. This might be particularly interesting in the light of the before-mentioned differing results of past studies on sensation seeking and disordered eating. The identification of potential moderators (e.g., emotional symptoms) could provide useful information on how sensation seeking can be characterized in the context of disordered eating and for future research on risk factors. Thus, the primary aim of the present study was to evaluate associations between sensation seeking and disordered eating, including eating disorder psychopathology and key behaviors of eating disorders in a large sample of healthy adolescents. Adding to previous literature, associations between sensation seeking and relevant sociodemographic variables (age, sex, and SES) were also examined. Despite contradictions in the reviewed literature, we hypothesized that sensation seeking would be positively associated with disordered eating, specifically restraint, overeating, loss of control, and binge eating. Additionally, we explored whether age- and sex-specific body mass index (BMI) and behavioral difficulties would moderate the association between sensation seeking and disordered eating. We expected stronger associations in adolescents with higher BMI and in those who showed more hyperactivity/inattention and emotional symptoms.

## Methods

### The LIFE child study

Data for the present study were collected between 2012 and 2017 for the LIFE Child study, a longitudinal study conducted at the Research Center for Civilization Diseases in Leipzig, Germany. Participants were children, parents, and pregnant women not suffering from chronic, chromosomal, or syndromal diseases, recruited via advertisements in health centers, schools, media, or word of mouth. LIFE Child evaluates child development from the 24th week of gestation until those children are 21 years of age, with a specific focus on the development of non-communicable diseases (e.g., obesity, allergies, and depression). For in-depth research on obesity, the LIFE Child Study includes a subcohort of children with obesity (in addition to a large cohort of normal weight children). Children are recruited until the age of 16 years and invited to attend annual follow-ups. The study program includes interviews, medical examinations, standardized tests, questionnaires, and the collection of biological samples. Study participants are informed about the study program, the long-term use of data, potential risks associated with participation, and their right to withdraw from the study. The LIFE Child study was designed to conform to the Declaration of Helsinki, and the study program was approved by the Ethics Committee of the University of Leipzig (Reg. No. 477/19-ek). Before enrolling participants, we obtained informed written consent from all parents as well as assent from children aged 12 or older [42, 43].

### Participants

Thirteen- to 15-year-old LIFE Child participants from both cohorts (normal weight and obesity) were included when information on sensation seeking, BMI, SES, behavioral difficulties, and disordered eating was available. If participants provided data at more than one time point, only the last one was included. Two participants were excluded due to invalid or implausible responses, leaving a final study sample of 400, with 190 (47.5%) male adolescents and 210 (52.5%) female adolescents.

### Measures

Body Mass Index (BMI): Trained research assistants measured the height and weight of participants. BMI (kg/m²) was calculated and transformed into BMI-standard deviation scores (BMI-SDS) on the basis of to age- and sex-adjusted percentiles [44, 45]. In addition, using the evidence-based (S3) guidelines of the Working Group on Childhood and Adolescent Obesity (AGA) from the German Obesity Society (DAG) and the German Society of Pediatrics and Adolescent Medicine (DGKJ) [45], adolescents´ weight status can be determined with BMI-SDS cutoff values. In the present study, adolescents were classified into two groups: the first having underweight (BMI-SDS < -1.28) or normal weight (-1.28 ≤ BMI-SDS ≤ 1.28; *n* = 305) and the second having overweight (1.28 < BMI-SDS ≤ 1.88) or obesity (BMI-SDS > 1.88; *n* = 95). Due to a low number of adolescents with underweight (*n* = 32) and no significant differences between adolescents with normal or underweight regarding the variables on sensation seeking, adolescents with underweight and normal weight were grouped together.

Socioeconomic status (SES): A family´s SES was determined by using an index (adapted to the “Winkler-Stolzenberg-Index”) that combines information on parental education (school and professional), occupational status, and household equivalent income [46]. Parents provided information on each of the three indicators, which were scored from 1 to 7. A sum score was derived with a minimum of 3 points and a maximum of 21. Higher scores indicate higher SES. Based on cut-offs created in a representative German sample, the composite score can be used to categorize a family’s SES as low (3.2–8.7 points), medium (8.8–16.9 points), or high (17.0–21.0 points) [47].

Arnett Inventory of Sensation Seeking (AISS-D): Sensation seeking was assessed with the revised version of the AISS, translated into German (AISS-D) [7, 9]. In the shortened 12-item version, adolescents were asked to identify the extent to which the behavior described them (1 = very well, 2 = somewhat, 3 = not very well, 4 = not at all) [7]. The revised AISS-D contains two subscales: a seven-item intensity subscale (IS) and a five-item novelty subscale (NS, referring to openness to experience. Scores on the single subscales are also combined into a mean total score (TS). In the present sample, the internal consistency (Cronbach´s alpha) values were 0.57 for IS, 0.63 for NS, and 0.64 for the TS (McDonald´s omega: 0.58, 0.65, and 0.65, respectively).

Eating Disorder Examination-Questionnaire for Children (ChEDE-Q): The child version (ChEDE-Q [48]) of the Eating Disorder Examination-Questionnaire (EDE-Q) [49] was applied to assess the specific psychopathology of eating disorders via self-report. It covers four subscales (restraint, eating concern, weight concern, shape concern), containing 22 items in total, which can also be combined into a global score. Each item can be answered on a 7-point Likert scale ranging from 0 (*no days/not at all*) to 6 (*every day/to an extreme degree*). The reference period is the past 28 days. In the present study, the internal consistency (Cronbach´s alpha) values were 0.85 for the restraint subscale, 0.73 for eating concern, 0.87 for weight concern, 0.92 for shape concern, and 0.95 for the ChEDE-Q global score (McDonald´s omega: 0.86, 0.73, 0.88, 0.93, and 0.95, respectively).

Diagnostically relevant symptoms of eating disorders were assessed with six additional items (episodes of overeating, loss of control [LOC], objective binge eating, self-induced vomiting, laxative misuse, driven exercising). Individuals were asked to indicate how many times or on how many days over the past 28 days these diagnostic symptoms of eating disorders occurred [48]. Due to a low number of adolescents reporting self-induced vomiting (*n* = 14) and laxative misuse (*n* = 6), these two items were excluded from evaluation.

Strengths and Difficulties Questionnaire (SDQ): The SDQ assesses behavioral strengths and difficulties in children and adolescents [50, 51]. For the present study, we used the self-report version of the SDQ. It consists of 25 items answered on a 3-point Likert scale (0 = *not true*, 1 = *somewhat true*, 2 = *certainly true*). The items form the five subscales hyperactivity/inattention (e.g., restlessness, distraction etc.), emotional symptoms (e.g., worries, somatic symptoms, feelings of unhappiness, fears etc.), conduct problems (e.g., fighting, lying, stealing etc.), peer problems (e.g., being lonely, bullied etc.), and prosocial behavior (e.g., being kind, helping out, sharing etc.). For each subscale, items are summed into a sum score ranging from 0 to 10, with higher scores indicating more difficulties/prosocial behavior [50]. The internal consistency (Cronbach´s alpha) values were 0.71 for the hyperactivity scale, 0.71 for emotional symptoms, 0.46 for conduct problems, and 0.64 for peer problems (McDonald´s omega: 0.71, 0.72, 0.49, and 0.66, respectively). For analysis, on the basis of Becker et al.´s results, adolescents were classified as having low to medium levels (“normal” to “borderline”) or high levels (“abnormal”) of behavioral difficulties [52]. The prosocial scale was not considered in our study.

### Statistical analysis

Descriptive statistics were given as the mean (standard deviation) for continuous variables with approximately symmetrical distributions, median (interquartile range) for continuous variables with highly skewed distributions, and counts (percentages) for discrete variables. Data were analyzed with R [53]. Multiple linear regression models were applied to assess associations and moderating effects. First, age (as a continuous measure), sex (female adolescents vs. male adolescents), BMI groups (adolescents with underweight or normal weight vs. adolescents with overweight or obesity), and SES (as a continuous measure) were included as independent variables, and the standardized values of the subscales (IS, NS) and the total score (TS) of the AISS-D were included as dependent variables. Second, associations between the standardized TS of the AISS-D (independent variable) and the different subscales and diagnostic-symptom items (as continuous measures) of the ChEDE-Q (dependent variables) were examined. All models were controlled for age, sex, and SES. Third, it was evaluated whether the associations between the standardized TS of the AISS-D and subscales, the global score, and diagnostic-symptom items (as continuous measures) of the ChEDE-Q were moderated by BMI groups, behavioral difficulties of the SDQ (high levels vs. low to medium levels), age (as a continuous measure), or sex. The strengths of the effects were indicated by *b* (unstandardized regression coefficient). For each association, the effect size index *f*^*2*^ was assessed (small effect size: *f*^*2*^ = 0.02; medium effect size: *f*^*2*^ = 0.15; large effect size: *f*^*2*^ = 0.35) [54]. Associations and interactions were considered statistically significant and were reported if the two-tailed p value was < 0.05. P-values were adjusted (p_adj) for multiple testing using the Benjamini–Hochberg method to control the false discovery rate (FDR). Interactions were retained if the variance inflation factor was < 5.

## Results

### Sample description

Table 1 presents sociodemographic and anthropometric characteristics of the sample and descriptive statistics of the ChEDE-Q and SDQ. The sample had a mean age of *M* = 14.70 years (*SD* = 0.57). Most adolescents had normal weight (*n* = 273, 68.3%) and met the criteria for a medium SES (*n* = 251, 62.8%).

**Table 1:** Distribution of sociodemographic characteristics, anthropometric characteristics, sensation seeking, disordered eating, and behavioral difficulties in the sample and separately for female and male adolescents

	Total sample (*N* = 400)	Female adolescents (*n* = 210)		Male adolescents (*n* = 190)
	M (SD) or *n* (%)	M (SD) or *n* (%)		M (SD) or *n* (%)
Age, years	14.70 (0.57)	14.72 (0.57)		14.67 (0.57)
SES total score, 3-21	12.82 (3.45)	12.60 (3.54)		13.06 (3.33)
Low SES	48 (12.0%)	32 (15.2%)		16 (8.4%)
Medium SES	251 (62.8%)	127 (60.5%)		124 (65.3%)
High SES	101 (25.3%)	51 (24.3%)		50 (26.3%)
BMI-SDS	0.39 (1.26)	0.51 (1.30)		0.27 (1.21)
Under-, normal weight	305 (76.3%)	153 (72.9%)		152 (80.0%)
Overweight, obesity	95 (23.8%)	57 (27.1%)		38 (20.0%)
Sensation seeking (AISS-D), possible range 1-4				
Intensity	2.54 (0.53)	2.42 (0.53)		2.67 (0.51)
Novelty	2.55 (0.63)	2.55 (0.64)		2.55 (0.63)
Total score	2.54 (0.45)	2.48 (0.45)		2.62 (0.44)
	Median (Q1-Q3)	Median (Q1-Q3)		Median (Q1-Q3)
Eating disorder psychopathology (ChEDE-Q), possible range 0-6				
Restraint	0.0 (0.0 - 0.6)	0.2 (0.0 - 1.0)		0.0 (0.0 - 0.4)
Eating concern	0.2 (0.0 - 0.6)	0.2 (0.0 - 1.2)		0.0 (0.0 - 0.4)
Shape concern	0.6 (0.1 - 1.9)	1.1 (0.3 - 2.7)		0.3 (0.0 - 1.0)
Weight concern	0.5 (0.0 - 2.0)	1.0 (0.0 - 2.8)		0.1 (0.0 - 1.0)
Global score	0.4 (0.1 - 1.4)	0.7 (0.2 - 1.9)		0.2 (0.0 - 0.7)
Diagnostic symptoms of eating disorders (ChEDE-Q), frequency of times/days over the past 28 days				
Overeating	1.0 (0.0 - 2.0)	0.0 (0.0 - 2.0)		1.0 (0.0 - 3.0)
Loss of control	0.0 (0.0 - 0.0)	0.0 (0.0 - 0.8)		0.0 (0.0 - 0.0)
Objective binge eating	0.0 (0.0 - 0.0)	0.0 (0.0 - 1.0)		0.0 (0.0 - 0.0)
Driven exercising	0.0 (0.0 - 2.0)	0.0 (0.0 - 2.0)		0.0 (0.0 - 3.0)
High levels (n, %)				
Behavioral difficulties (SDQ), possible range 0-10				
Hyperactivity (7 - 10)	33, 8.3%	13, 6.2%	20, 10.5%	
Emotional symptoms (6 - 10)	39, 9.8%	32, 15.2%	7, 3.7%	
Conduct problems (5 - 10)	16, 4.0%	3, 1.4%	13, 6.8%	
Peer problems (5 - 10)	40, 10.0%	25, 11.9%	15, 7.9%	

*M* mean, *SD* standard deviation, *SES* socioeconomic status, *BMI-SDS* BMI-standard deviation scores, *AISS-D* Arnett Inventory of Sensation Seeking, German version, *ChEDE-Q* Eating Disorder Examination-Questionnaire for Children, *SDQ* Strengths and Difficulties Questionnaire

### Associations between sensation seeking and age, sex, SES, and weight status (BMI groups)

Table 2 presents the associations between sensation seeking and age, sex, SES, and BMI groups.

Male adolescents showed significantly higher TS (b = 0.30, *p* = .002, p_adj = 0.02) and IS (b = 0.46, *p* < .001, p_adj < 0.001) than female adolescents. NS was significantly positively associated with SES (b = 0.06, *p* < .001, p_adj < 0.001). IS, NS, and TS were not significantly associated with age and did not differ between BMI groups (overweight and obesity vs. underweight and normal weight).

### Associations between sensation seeking and eating disorder psychopathology

Table 3 presents associations between sensation seeking and eating disorder psychopathology.

A significantly positive association between TS and ChEDE-Q restraint (b = 0.13, *p* = .008, p_adj = 0.03) was observed. Positive associations were observed between TS and eating concern (b = 0.09, *p* = .02), and the global score (b = 0.12, *p* = .02), but these associations did not remain statistically significant after correction for multiple testing (p_adj: 0.07, 0.08).

Regarding the diagnostic-symptom items of the ChEDE-Q, TS was significantly associated with more episodes of overeating (b = 0.78, *p* < .001, p_adj < 0.01). Positive associations between TS and LOC eating episodes (b = 0.30, *p* = .05), and binge-eating episodes (b = 0.25, *p* = .03) did not remain statistically significant after correction for multiple testing (p_adj: 0.14, 0.09).

**Table 2:** Associations between sensation seeking (revised AISS-D, standardized IS, NS, and TS) and age (as a continuous measure), sex (male vs. female adolescents), SES (as a continuous measure), BMI groups (overweight and obesity vs. underweight and normal weight)

	b [95% CI]	f^2^	*p*	*p* (adjusted)
Age				
Intensity seeking	0.03 [-0.13, 0.20]	0.06	0.69	0.80
Novelty seeking	-0.01 [-0.18, 0.16]	0.05	0.87	0.90
Total score	0.02 [-0.15, 0.19]	0.03	0.86	0.90
Sex (reference = female)				
Intensity seeking	0.46 [0.27, 0.66]	0.06	< 0.001***	< 0.001***
Novelty seeking	-0.03 [-0.22, 0.17]	0.05	0.78	0.85
Total score	0.30 [0.10, 0.50]	0.03	0.002**	0.02*
SES				
Intensity seeking	-0.01 [-0.04, 0.01]	0.06	0.30	0.48
Novelty seeking	0.06 [0.03, 0.09]	0.05	< 0.001***	< 0.001***
Total score	0.03 [-0.002, 0.05]	0.03	0.07	0.18
BMI groups (reference = underweight and normal weight)				
Intensity seeking	0.03 [-0.20, 0.27]	0.06	0.78	0.85
Novelty seeking	-0.04 [-0.28, 0.20]	0.05	0.76	0.85
Total score	0.002 [-0.24, 0.24]	0.03	1.00	1.00

*b* unstandardized regression coefficient, *CI* confidence interval, *f*^*2*^ effect size index, all associations are adjusted for age (as a continuous measure), sex, and socioeconomic status (as a continuous measure)

**Table 3:** Associations between sensation seeking (revised AISS-D, standardized TS) and disordered eating (subscales and diagnostic-symptom items of the ChEDE-Q)

Disordered eating (ChEDE-Q)	b [95% CI]	f^2^	*p*	*p* (adjusted)
Restraint ^c, d^	0.13 [0.04, 0.23]	0.06	0.008**	0.03*
Eating concern ^c, d^	0.09 [0.02, 0.17]	0.09	0.02*	0.07
Weight concern ^a, c,d^	0.13 [-0.02, 0.28]	0.12	0.10	0.24
Shape concern ^c, d^	0.13 [-0.01, 0.27]	0.11	0.07	0.18
Global score ^c, d^	0.12 [0.02, 0.23]	0.12	0.02*	0.08
Overeating	0.78 [0.36, 1.20]	0.04	< 0.001***	< 0.001***
Loss of control ^b, c^	0.30 [0.005, 0.59]	0.02	0.05*	0.14
Objective binge eating ^b, c^	0.25 [0.03, 0.47]	0.02	0.03*	0.09
Driven exercising	0.31 [-0.26, 0.88]	0.01	0.29	0.47

*b* unstandardized regression coefficient, *CI* confidence interval, *f*^*2*^ effect size index, all associations are adjusted for age (as a continuous measure), sex, and socioeconomic status (as a continuous measure)

^a^ Significant interaction with BMI groups: stronger association in adolescents with overweight and obesity compared with adolescents with underweight and normal weight. ^b^ Significant interaction with hyperactivity/inattention: stronger association with increasing hyperactivity/inattention. ^c^ Significant interaction with emotional symptoms: stronger association with increasing number of emotional symptoms. ^d^ Significant interaction with peer problems: stronger association with increasing number of peer problems

### Moderating effects of weight status (BMI groups) and behavioral difficulties (high vs. low to medium)

**Fig. 1:**
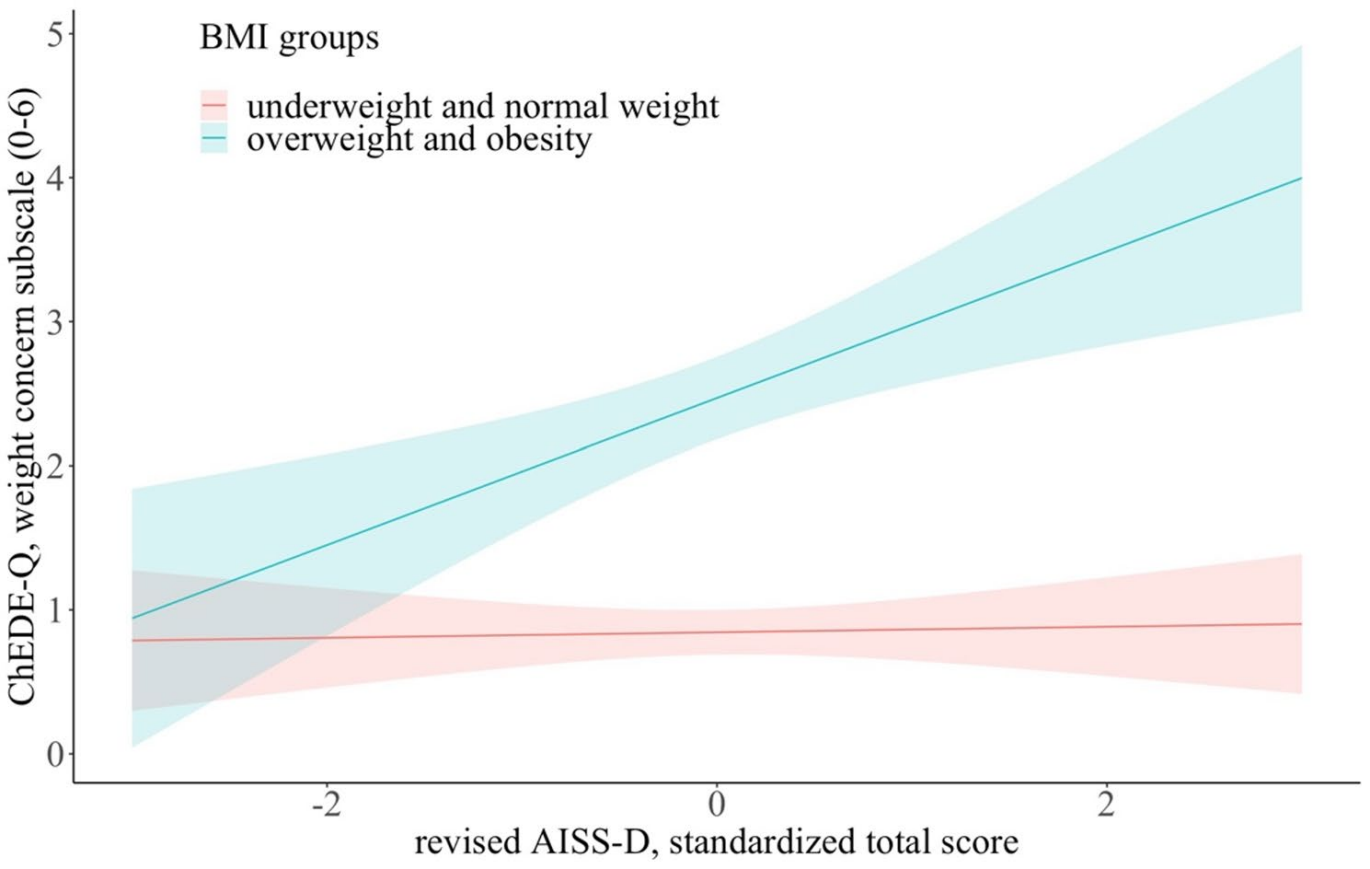
Effect plots illustrating the weight concern subscale of the ChEDE-Q (and 95% confidence interval) depending on the standardized TS of the revised AISS-D for adolescents with underweight and normal weight (red regression line) and adolescents with overweight and obesity (blue regression line)

Regarding weight concern, the analyses revealed a significant interaction between BMI group and sensation seeking TS. Whereas the associations between TS and the weight concern subscale was only weak and nonsignificant in adolescents with underweight and normal weight, it was significant and positive in adolescents with overweight and obesity (b = 0.54, f^2^ = 0.20, *p* = .005, p_adj = 0.02). Regarding shape concern and the ChEDE-Q global score, interaction effects did not remain statistically significant after correction for multiple testing (*b*, *f*^*2*^, *p*,* p_adj*: shape concern: 0.40, 0.12, 0.02, 0.10; global score: 0.34, 0.14, 0.008, 0.07). Figure 1 illustrates how BMI group moderated the association between the TS and the weight concern subscale of the ChEDE-Q.

**Fig. 2:**
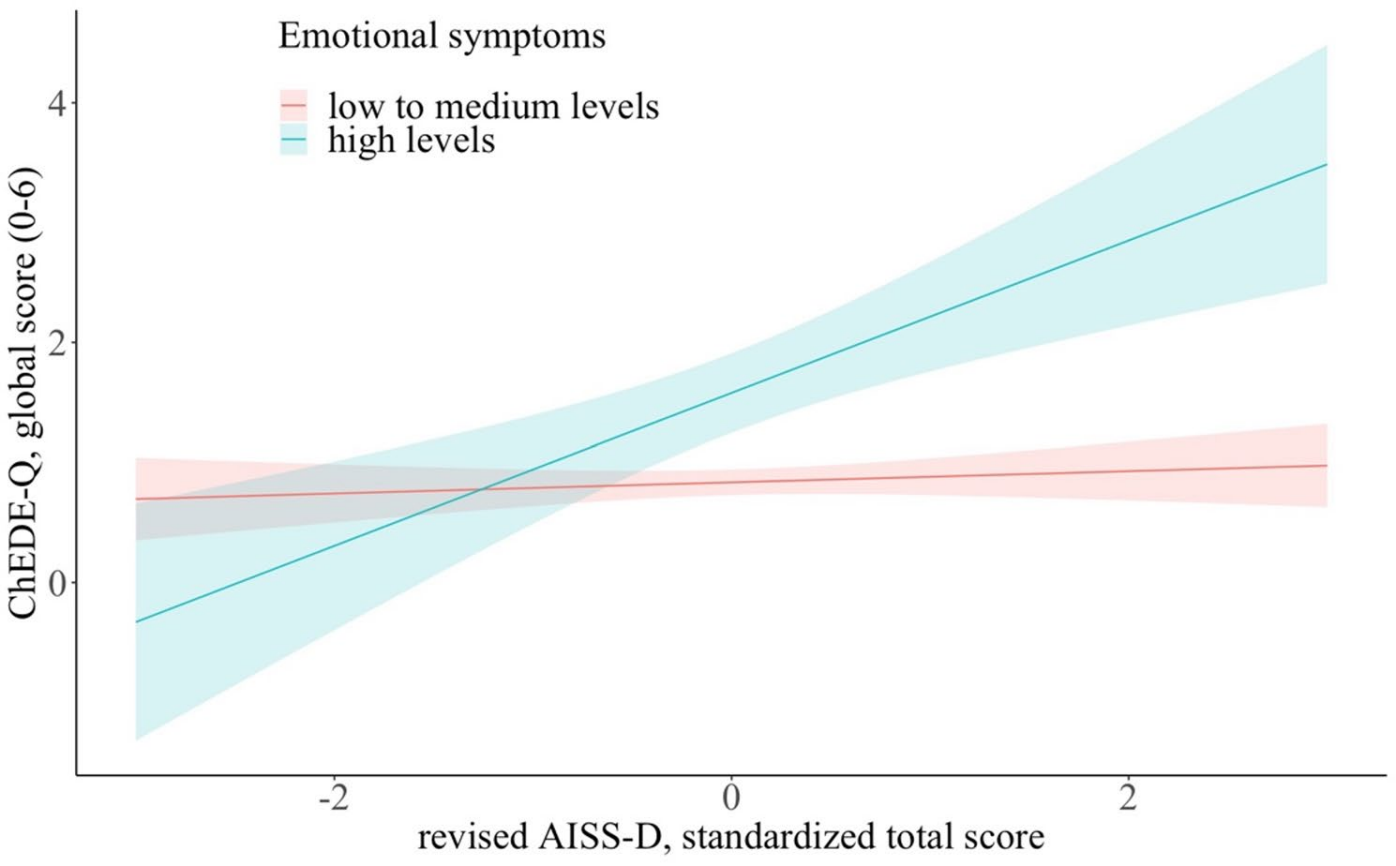
Effect plots illustrating the ChEDE-Q global score (and 95% confidence interval) depending on the standardized TS of the revised AISS-D for adolescents with low to medium levels of emotional symptoms (red regression line) and adolescents with high levels of emotional symptoms (blue regression line)

In addition, associations between TS and some subscales of the ChEDE-Q were significantly moderated by the levels of emotional symptoms (*b*, *f*^*2*^, *p*,* p_adj*: restraint: 0.56, 0.10, < 0.001, < 0.01; eating concern: 0.50, 0.19, < 0.001, < 0.001; weight concern: 0.72, 0.18, 0.003, 0.02; shape concern: 0.60, 0.19, 0.007, 0.03; global score: 0.59, 0.20, < 0.001, < 0.01) and levels of peer problems (*b*, *f*^*2*^, *p p_adj*: restraint: 0.38, 0.08, 0.007, 0.03; eating concern: 0.29, 0.14, 0.01, 0.04; weight concern: 0.60, 0.16, 0.006, 0.03; shape concern: 0.63, 0.17, 0.002, 0.01; global score: 0.49, 0.18, 0.001, < 0.01). In adolescents with high levels of emotional symptoms or peer problems, the associations between TS and the abovementioned subscales or the ChEDE-Q global score were significant and positive, whereas no significant association could be shown for low to medium levels of emotional symptoms or peer problems. Figure 2 shows how the association between TS and the ChEDE-Q global score was moderated by emotional symptoms.

Sensation seeking´s associations with LOC eating and binge eating were significantly moderated by hyperactivity/inattention (*b*, *f*^*2*^, *p*,* p_adj*: LOC eating: 1.80, 0.06, 0.003, 0.02; binge eating: 1.20, 0.05, 0.008, 0.03) and emotional symptoms (*b*, *f*^*2*^, *p*,* p_adj*: LOC eating: 2.01, 0.09, < 0.001, < 0.001; binge eating: 1.30, 0.07, < 0.001, < 0.01). Associations between TS and these diagnostic symptoms of eating disorders were significant and positive in adolescents with high levels of hyperactivity/inattention and emotional problems and nonsignificant in those with low to medium levels.

The strengths of the associations between TS and the subscales, the global score, and the diagnostic-symptom items of the ChEDE-Q did not vary depending on the levels of conduct problems, age, or sex.

## Discussion

The present study aimed to assess associations between sensation seeking and disordered eating and how weight status and behavioral difficulties moderated these associations in a large sample of adolescents, taking into account relevant sociodemographic variables. As expected, positive associations between sensation seeking and overeating were identified. Unexpectedly, associations between sensation seeking and loss of control (LOC) eating, as well as binge eating did not remain significant after correction for multiple testing. Concerning eating disorder psychopathology, higher sensation seeking was significantly related to higher restraint. Furthermore, a higher BMI and more symptoms of hyperactivity, emotional symptoms and peer problems amplified the association between sensation seeking and disordered eating.

Past research on associations between personality traits and disordered eating was often conducted on treatment-seeking samples of patients with eating disorders. Due to the higher prevalence and persistence of eating disorders in female individuals [55, 56], male participants were rarely included in these investigations. In the present study, which included a large sample of adolescents from the community balanced for sex, our findings suggest no sex differences in the association between sensation seeking and disordered eating, indicating that female adolescents and male adolescents with higher sensation seeking levels might have similar underlying conditions or motivations to engage in unhealthy eating behaviors.

### Sensation seeking and sociodemographic and anthropometric variables

We found higher total levels of sensation seeking (TS) in male adolescents than in female adolescents. However, in line with previous studies [7, 9, 57], these differences were mainly driven by higher intensity seeking (IS) in male adolescents, whereas there was no sex-related difference for novelty seeking (NS) [58]. Even though sex differences in sensation seeking have remained mostly stable since the late 1970s [59], they may still reflect socialization rather than a biological predisposition or, most likely, a combination of the two. Hence, they should be reviewed critically. In contrast to previous findings referring to individuals aged 10 to 30 years [3, 60], the study did not demonstrate that sensation seeking levels increased with age during adolescence, a finding that might be attributable to the smaller age range used in the present study. In line with other studies [4, 10, 11], the present findings did not reveal a significant relationship between the sensation seeking total score and SES. However, higher novelty seeking was associated with a higher SES, which may be because financial and educational opportunities help create a stimulating environment that is safe for exploring.

### Sensation seeking and disordered eating

As expected, higher sensation seeking was significantly related to overeating (small effect size). Unexpectedly, positive associations between sensation seeking and LOC eating, as well as between sensation seeking and binge eating did not remain significant after multiple correction. These findings are in line with previous studies that found no significant association between sensation seeking and binge eating in college students [61], nor did they find a significant association between sensation seeking and eating disorder psychopathology in female university students enrolled in a psychology class [62]. In contrast, other studies found higher sensation seeking levels in young women (from clinical [26] and nonclinical populations [27]) with binge eating symptoms compared with control groups [26, 27]. Importantly, in most of these studies [27, 61, 62], participants were 17 years of age or older. Since present associations were drawn from a nonclinical sample of adolescents, the relatively small number of those reporting episodes of LOC eating or binge eating might have contributed to loss of significance. Overeating as compared to LOC- or binge eating, refers to the consumption of an unusually large amount of food, but does not include an accompanied feeling of LOC. Adolescents with high sensation seeking levels who overeat might show underlying motivations, such as feelings of reward, which potentially outweigh negative feelings, such as LOC. Apart from that, positive associations between sensation seeking and overeating might be rooted in difficulties in self-regulation in individuals with higher levels of the trait. This idea is supported by a large MRI study in healthy young adults (18–35 years), which identified links between increased sensation seeking and reduced cortical thickness in brain areas involved in the cognitive control circuitry [63]. Thus, impairments in self-regulation in individuals with higher sensation seeking levels might not be solely attributable to developmental processes during adolescence. In line with the dual-system model [64], findings by Van Malderen et al. suggest that weakened regulatory processing (e.g., inhibitory control) along with heightened reactive processing (e.g., reward responsiveness) significantly increased the likelihood of experiencing LOC eating in adolescents from the general population [65]. However, to our knowledge, this has not yet been confirmed for overeating too.

In previous studies, sensation seeking has often been examined in the context of connections between impulsivity and disordered eating. A frequently used questionnaire to assess impulsivity is based on the assumption that impulsivity is a multi-faceted construct consisting of negative urgency, lack of premeditation, lack of perseverance, and sensation seeking (UPPS) [66]. Consequently, sensation seeking can be acknowledged as a dimension of impulsivity, but is not the same construct. In the present study, analyses revealed significant positive associations between sensation seeking and eating disorder psychopathology, particularly the restraint and eating concern scales. Restraint comprises the intention to follow restrictive dietary rules but not necessarily the absolute restriction of caloric intake [67]. Previous findings suggest that high-restraint eaters tend to overeat when they are also more impulsive [68]. If that would apply similarly for sensation seeking, then restraint thinking in adolescents with greater sensation-seeking behavior may express an attempt, rather than a successful realization, to compensate for the potential consequences of overeating, such as weight gain. The association between sensation seeking and a negative body image (i.e., the weight and shape concern scales) was not significant, possibly explainable by the usual, development-related increase in both sensation seeking during adolescence [3] and the prevalence of clinically relevant body dissatisfaction during adolescence [69].

Unlike previous findings [33, 34, 70], our results did not indicate a significant relationship between weight status and sensation seeking, a finding that may be related to methodological differences, for example, the use of a different self-report questionnaire to assess sensation seeking (UPPS-P). However, in adolescents with obesity and overweight (but not in adolescents with normal or underweight), the association between sensation seeking and weight concern was significant and positive, with moderate effect sizes. Previous findings indicate a significantly lower body esteem in children and adolescents with overweight or obesity compared to those with normal weight [71]. Adolescents with overweight or obesity who show higher levels of sensation seeking may be more vulnerable to stigmatization and discrimination than those with low levels of sensation seeking, and therefore internalize body dissatisfaction more strongly.

As hypothesized, hyperactivity moderated the association between sensation seeking and disordered eating. In adolescents who showed few or no symptoms of hyperactivity, we observed no associations between sensation seeking and restraint, LOC eating, or binge eating, whereas, in adolescents with a higher hyperactivity score, these associations were significant and positive. Neural correlates of pediatric attention-deficit/hyperactivity disorder (ADHD) and LOC or binge eating were previously discussed in a review indicating overlaps in reward-, response inhibition-, and emotional regulation circuits [72]. Findings of Seitz et al. indicate more impulsivity in female patients with bulimia nervosa (aged 15–35 years) who also had former childhood ADHD compared with those without childhood ADHD [73]. This might be similar in individuals with higher sensation seeking levels who show symptoms of disordered eating. According to the vigilance regulation model of ADHD, sensation seeking and hyperactivity may serve as regulatory attempts to stabilize vigilance (meaning central nervous arousal) [74]. Hyperactivity might help to create an optimal level of stimulation in adolescents with greater sensation seeking. On the other hand, it might reinforce uncontrolled eating behavior to compensate for a lack of external stimuli.

As expected, more emotional symptoms, such as general worries and feelings of unhappiness, strengthened the association between sensation seeking and disordered eating. This finding was true for eating, weight, and shape concerns (moderate effect sizes) as well as for restraint, LOC eating, and binge eating (small effect sizes). Affect regulation plays an important role in eating disorders, specifically those characterized by binge- and purge-type behavior [75, 76]. Findings by Schaefer et al. demonstrated increased negative affect in treatment-seeking adults (18 to 64 years) who met the criteria for DSM–5 BED prior to binge-eating episodes and improvement in negative affect immediately after binge eating episodes. Their negative affect was alleviated immediately after binge eating episodes, mostly related to the sense of guilt [76]. Moreover, negative urgency, the tendency to act impulsively when experiencing negative affect [66], was related to cognitive (e.g., dietary restraint, weight and shape concerns) and behavioral symptoms (e.g., binge eating and emotional eating) of eating disorders in girls [77]. Referring to sensation seeking, as a construct related to impulsivity, negative emotional symptoms might display an amplifier of unhealthy or risky behavior. Longitudinal findings demonstrated correlations between changes (increases and decreases) in sensation seeking and negative urgency measures in 13- to 16-year-olds [78], indicating that this age might be an especially vulnerable phase. In summary, despite potential negative outcomes, adolescents with high sensation-seeking levels who experience negative affect might engage in activities that satisfy short-term feelings of reward and rapidly create appealing sensations, such as overeating or binge eating, to compensate for uncomfortable sensations and alleviate emotional distress.

Peer problems were also shown to strengthen the association between sensation seeking and disordered eating (restraint; eating, weight, and shape concerns). Amongst other things, adolescents with high levels of peer problems reported being bullied or feeling lonely more often than those with low levels. Feelings of loneliness or being disrespected are likely emotionally challenging. Therefore, a moderating effect of peer problems might have similar underlying reasons as those mentioned above for emotional symptoms.

Sensation seeking is a personality trait, and it is important not to pathologize this trait, especially in light of previous studies that have focused primarily on maladaptive behavior. For this reason, in a clinical context, it may be useful to examine and target potentially relevant behaviors and affective states (e.g., hyperactivity and emotional symptoms) in adolescents with high levels of sensation seeking who show symptoms of disordered eating. Future studies could investigate, whether there exists a causal relationship between sensation seeking, behavioral difficulties, such as emotional symptoms, and disordered eating.

### Strengths and limitations

The present study was conducted in a large sample of adolescents, balanced for sex, which enabled us to investigate potential sex differences. We used established self-report questionnaires and objective anthropometrics. By applying the ChEDE-Q, we were able to assess not only behavioral features of eating disorders, but also eating disorder psychopathology, including e.g., restraint and body dissatisfaction, in adolescents. However, the cross-sectional design allowed us to investigate only associations but not causal relationships between the variables. Although the sample was balanced for sex, there was no information on adolescents´ gender identity which should be considered in future studies. The overrepresentation of participants with high parental SES did not accurately represent the distribution in the general German population [46]. Furthermore, analyses were performed with a non-clinical sample with a low frequency of eating disorder symptoms. Subgroup analyses were conducted with relatively small sample sizes. Therefore, the interpretation of non-significant associations or interactions should be exercised with caution. Finally, the low internal validity of the AISS must be considered, even though the value was comparable to previous studies [9, 58, 79]. Results of Roth et al. indicate acceptable validity of the AISS-D and support for the proposed two-factor structure of sensation seeking in adolescents [9]. On a conceptual level, it should be noted that sensation seeking is not a homogenous construct but rather a personality trait that can manifest itself in different ways. This is reflected, at least in part, in the items of the AISS-D and could contribute, among other things, to the low internal consistency of the questionnaire. Future research should examine how the questionnaire can be modified (e.g., addition or deletion of items) to improve the internal consistency. Similarly, this caution applies to the conduct and peer problems subscales from the SDQ.

## Conclusion

The present study revealed a positive association between sensation seeking and disordered eating in adolescents, specifically overeating and restraint. In individuals with a higher BMI, more hyperactivity, and more internalizing symptoms, we observed a stronger link between sensation seeking and disordered eating. These moderating effects suggest that therapeutic interventions addressing hyperactivity and enhancing emotion regulation might be especially beneficial for those prone to impulsive behaviors, such as sensation seeking. Hence, future longitudinal studies should consider potential moderators, such as negative urgency, in understanding the role of sensation seeking in the development of disordered eating during adolescence.

## Data Availability

The datasets generated and/or analyzed during the current study are not publicly available due to ethical restrictions. The LIFE Child study is a study collecting potentially sensitive information. Publishing data sets is not covered by the informed consent provided by the study participants. Furthermore, the data protection concept of LIFE requests that all (external as well as internal) researchers interested in accessing data sign a project agreement. Researchers that are interested in accessing and analyzing data collected in the LIFE Child study may contact the data use and access committee (forschungsdaten@medizin.uni-leipzig.de).

## Abbreviations

AISS-D: Arnett inventory of sensation seeking (German version)
ChEDE-Q: Eating disorder examination-questionnaire for children
SDQ: Strengths and difficulties questionnaire
BMI: Body mass index
BMI-SDS: Body mass index- standard deviation scores
SES: Socioeconomic status
TS: AISS-D total score
IS: AISS-D intensity subscale
NS: AISS-D novelty subscale
LOC: loss of control (ChEDE-Q)
